# Potential Effects of the Different Matrices to Enhance the Polyphenolic Content and Antioxidant Activity in Gluten-Free Bread

**DOI:** 10.3390/foods12244415

**Published:** 2023-12-08

**Authors:** Carolina Bueno, Roberta Thys, Bruna Tischer

**Affiliations:** Food Science and Technology Institute, Federal University of Rio Grande do Sul, Av. Bento Gonçalves, 9500, Porto Alegre 91501-970, RS, Brazil; vbueno.carolina@gmail.com (C.B.); bruna.tischer@ufrgs.br (B.T.)

**Keywords:** gluten-free, plant-based matrices, antioxidants, functional bread, bioactive compounds

## Abstract

Gluten-related disorders, including celiac disease, wheat allergy, and non-celiac gluten sensitivity, have emerged as a significant phenomenon affecting people worldwide, with an estimated prevalence of nearly 5% globally. The only currently available treatment for this disease involves the exclusion of gluten from the diet, which is particularly challenging in the case of bakery products. Gluten-free bread (GFB) presents certain disadvantages when compared to traditional wheat bread, including inferior sensory attributes, technological characteristics, and lower protein and fiber content. Numerous studies have focused on strategies to improve these aspects of GFB. However, there are limited reviews regarding the content of the bioactive compounds of GFB, such as polyphenols. Polyphenols are molecules found in various foods that play a vital role in protecting the body against oxidative stress. This is particularly relevant for individuals with gluten intolerance or celiac disease, as they often experience increased oxidative stress and inflammation. Therefore, the objective of this review is to explore the use of different strategies for increasing the polyphenolic content and the antioxidant properties of GFB. Gluten-free cereals and pseudocereals are the most used matrices in GFB. Buckwheat can be a valuable matrix to enhance the nutritional profile and antioxidant properties of GFB, even more so when the whole grain is used. In the same way, the addition of various by-products can effectively increase the bioactive compounds and antioxidant activity of GFB. Furthermore, regarding the contribution of the phenolics to the bitterness, astringency, color, flavor, and odor of food, it is essential to analyze the sensory properties of these breads to ensure not only enriched in bioactive compounds, but also good consumer acceptance. In vitro studies are still in few number and are very important to execute to provide a better understanding of the bioactive compounds after their consumption.

## 1. Introduction

Celiac disease is an autoimmune disorder that primarily affects the small intestine. It is triggered by the consumption of gluten, a protein found in wheat, barley, and rye. For these individuals, the consumption of gluten causes an immune response that damages the small intestine, specifically the villi. Besides individuals diagnosed with celiac disease, a portion of the population experiences non-celiac wheat/gluten sensitivity, which leads them to suffer symptoms resembling those of celiac disease upon consuming gluten-containing food products The only treatment for these diseases is a strict gluten-free diet, meaning the necessity to avoid all foods containing gluten. Allied with this, there is a growing interest in the population in general to the gluten-free diet, increasing the interest in gluten-free products [1,2].

Many scientific works have sought to identify the best ways to produce gluten-free bread (GFB) with sensorial and technological quality. The low protein content in GF flours and the absence of the gluten network, responsible for the gas retention and structure of bread are the main factors that contribute to the challenge [3]. Apart from the challenge in production technicality, the low content of proteins, vitamins (folic acid, B vitamins), dietary fibers, minerals (Fe, Ca, Mg, Cu), and phenolic compounds are the most important deficiencies in GFB, since in these products whole cereal grains are often excluded [4,5,6,7]. Although a lot of research has been conducted to improve the nutritional value of GFB, nowadays, the focus is put on its bioactive compounds.

Bioactive compounds are phytochemicals found in food matrices (mainly fruits and vegetables), which are capable of modulating metabolic processes and promoting health benefits. Among these benefits, it is possible to mention the antioxidant capacity, inhibition or induction of enzymes, and induction and inhibition of gene expression, all of which results in a reduction of the risk of developing cardiovascular disease, diabetes, obesity, and cancer. The most common bioactive compounds found in food matrices are polyphenols, terpenoids, glucosinolates, and sulfur compounds [7]. 

In particular, polyphenols exhibit a wide range of physiological properties, such as antiallergenic, antiatherogenic, anti-inflammatory, antimicrobial, antioxidant, antithrombotic, cardioprotective, and vasodilatory effects [7]. This is very relevant for individuals with gluten intolerance or celiac disease, as they often experience high oxidative stress and inflammation, which could be minimized by the use of antioxidants in the diet [8]. However, during breadmaking, these compounds could be affected by the temperature. The type of heated subtracts and processing conditions are the main factors of the polyphenol loss [9]. Therefore, the changes in antioxidative properties of the final GFB need to be always evaluated.

The present review explores the use of different vegetable matrices to enhance the polyphenol content and antioxidant capacity in GFB. Owing to advancements in chemical techniques, various methods now exist for extracting, purifying, measuring, and identifying polyphenols. The extraction strategies employed depend on the specific type of polyphenol and its natural source. Consequently, this review refrains from making quantitative comparisons among the diverse findings.

## 2. Method

This review included studies that investigated strategies that attempt to improve the bioactive compound content in gluten-free bread. The databases of peer-reviewed literature Science Direct and Web of Knowledge were used. The descriptors used were “gluten-free bread”, “antioxidant”, and “total phenolic content”. The years of publication selected were in the range from 2010 up to 2021 and the restriction was English language. Specific exclusion criteria were as follows: (a) studies that did not present any of the targeted analysis made in the bread samples (after the breadmaking process), (b) studies that presented other products as samples (such as pasta, muffins, or biscuits), and (c) review papers.

After using the descriptors to search the studies, these were selected after reading the “bread making” section of materials and methods and verifying if the bread produced was in fact gluten-free. After that, the analysis methods performed were evaluated, and the studies that did not present any of the analyses targeted (ORAC, FRAP, DPPH, Total phenol content) were excluded. The last scan was to make sure that the targeted analyses were made using the bread samples, and not only in the food matrices, excluding the latter ones. With those steps, the present work ended up with 37 studies. The table showing all studies analyzed, the main analyses and the main results is present in Appendix A. 

## 3. Discussion

This chapter summarizes the different matrices used to enhance the polyphenolic content and antioxidant activity in GFB. The selected studies used the spectrophotometric method, based on the Folin–Ciocalteu assay, to determine the total phenolic content (TPC) of the GFB. 

The antioxidant capacity measurement methods can be divided into two main groups: in vitro and in vivo methods. For the in vitro methods, it is possible to classify them into two groups, according to the chemical reaction that happens between the bioactive compound and the free radical: the ones based on hydrogen atom transfer reactions and the ones based on electron transfer reactions. ORAC (oxygen radical absorbance capacity) and TRAP (total antioxidant potential) are examples of hydrogen atom transfer reactions, while DPPH (2,2-diphenyl-1-picryl-hydrazyl-hydrate) and FRAP (ferric reducing antioxidant power) are examples of electrons transfer reactions [10]. 

### 3.1. Acorn Flour

Acorn flour presented a high content of fat, particularly monounsaturated and polyunsaturated (oleic and linoleic acids). Additionally, the flour contained a high concentration of minerals, with potassium being particularly prominent. In terms of its phenolic profile, several compounds were identified, including rutin, catechin, ellagic acid, gallic acid, and syringic acid. One of the distinctive characteristics of acorns is their bitter taste, which is largely attributed to the presence of tannins. However, when it comes to Holm oak acorns, they have a slightly sweeter flavor compared to other oak varieties, thanks to their lower levels of tannins [11,12,13,14,15,16,17,18,19]. 

Used in an attempt to increase the phenolic compounds of GFB, acorn flour contains high levels of lipids, sterols, and phenolic compounds. Skendi et al. [20] substituted the original flour mix (rice/corn, 1:1) with acorn flour (Holm oak) in three different percentages to the original recipe: 5%, 15%, and 25%; they also tested three different hydration percentages: 65%, 70%, and 75% water content. For this study, the water content did not affect the TPC analysis of the different samples, but the addition of acorn flour increased the content of these substances. The higher the percentage substitution, the higher the TPC of GFB, with values ranging from 11.06 to 17.12 mg GAE/g DM.

Acorn flour (Holm oak) was also used in a study by Martins et al. [12], substituting 23% and 35% of the original flour/starch mix, which consisted of 46% buckwheat flour, 31% rice flour, and 23% potato starch. The acorn flour percentages used were defined by the replacement of buckwheat flour, being 23% equivalent to 50% substitution, and 35% equivalent to 75% substitution. The samples have presented an increase in the TPC when compared with the control bread, which presented 0.395 mg GAE/g DM, while bread with 23% presented 0.613 mg GAE/g DM and bread with 35% presented 0.848 mg GAE/g DM. Even with a higher percentage of addition of acorn flour, the TPC of the samples in this study is lower than in the study by Skendi et al. [20]. 

For the DPPH analysis (results expressed in mmol TE/100 g DM), the results showed a significantly higher scavenging activity for both acorn flour GFB in comparison with the control bread, but without difference from each other. For FRAP analysis, both samples presented higher results in comparison with the control bread. The values found were 0.041 mmol and 0.064 mmol TE/g DM for bread with 23% and A35%, respectively. The lowest addition of acorn flour caused an increase of nearly 6 times the value of the control bread, which is 0.007 mmol TE/g DM. The authors also observed an increment in the ABTS radical scavenging activity when the acorn flour was added, but without significant difference among the treatments [11].

### 3.2. Buckwheat Flour (BF) 

Buckwheat is a pseudocereal that contains high-quality proteins and lipids and a high content of minerals and dietary fiber [21]. Besides its high-quality proteins, buckwheat is also rich in many rare components that have healing effects on some chronic diseases. Among these components, the most attractive ones are flavones, flavonoids, phytosterols, d-chiro-Inositol, and myo-inositol. The flavonoids present in buckwheat include rutin, orientin, vitexin, quercetin, isoorientin, and isovitexin. Buckwheat varieties can contain anywhere between 12.6 and 35.9 mg of rutin of dry weight [17]. 

Sakac et al. [22] tested the use of BF in its light (LBF) and whole (WBF) forms to make GFB. The samples consisted of rice flour and LBF or WBF, with three different substitution percentages (10%, 20%, and 30%), resulting in six samples (rice flour:BF): 90:10 LBF, 80:20 LBF, 70:30 LBF, 90:10 WBF, 80:20 WBF, and 70:30 WBF.

The TPC increased with a higher addition of buckwheat flour, in both forms, and the WBF had higher quantities of these compounds. The results ranged from 0.35 to 0.95 mg GAE/g DM for LBF, and from 0.5 to 1.15 mg GAE/g DM for WBF. This study also analyzed the TPC of the buckwheat flour before baking, and the comparison between the flour and the bread showed that bread made with LBF had a higher impact compared to bread made with WBF [22].

The results for the DPPH analysis were expressed as IC_50_. The samples that had the addition of buckwheat flour showed a higher scavenging activity than the control (rice flour sample), and the 70:30 WBF sample had the highest value. In this study, the authors determined the yield of the IC_50_ values observed. The percentage of yield increased with the addition of buckwheat flour, and it had a greater impact on WBF. This is explained by the authors as a result of heat processing, which releases phenolic compounds from the cell walls of the grains. The use of WBF caused a higher impact on the chelating activity of Fe^2+^ compared to LBF. For the antioxidant capacity, the authors used the β-Carotene bleaching analysis and calculated the degradation rates according to the first-order kinetics using the equation by Al-Saikhan, Howard, and Miller. The results were expressed in IC_50_ value. Bread with higher quantities of buckwheat flour showed an increase in antioxidant capacity, with bread made using whole buckwheat flour having a greater impact compared to the use of light buckwheat flour. 

Alvarez-Jubete et al. [9] used BF made with and without the sprouting process. The control GFB was made using a gluten-free flour mix (without buckwheat). This flour mix was substituted by 50% regular buckwheat flour or 100% sprouted buckwheat flour. Both samples had higher concentrations of TPC compared to the GF control bread (without buckwheat), which had only 0.088 mg GAE/g DM. In contrast, bread with 50% buckwheat flour had 0.645 mg GAE/g DM and bread with the 100% of sprouted buckwheat flour had 1.16 mg GAE/g DM. These values are lower, and indicate that the breadmaking process causes the degradation of these compounds. Comparing the TPC of the buckwheat seeds (3.23 mg GAE/g DM) with sprouted buckwheat flour (6.70 mg GAE/g DM), it can be seen that the sprouting process is a good strategy to enhance the TPC of GFB. 

The authors also evaluated the antioxidant capacity using the FRAP analysis of breads containing 50% BF and 100% SBF. The results showed a significant increase in antioxidant capacity with the use of both described flours compared to the control bread. The values were 0.148 and 0.264 mg TE/g DM for 50% BF and 100% SBF, respectively, while the control bread showed 0.0476 mg TE/g DM. The FRAP analysis conducted by Costantini et al. also showed an increase in antioxidant capacity compared to the control bread, in this case, wheat bread. The common buckwheat bread had an antioxidant capacity of 430 mmol Fe^2+^ E/100 g DM and the Tartary buckwheat bread, 3 mol Fe^2+^ E/100 g DM. The control sample had only 60 mmol Fe^2+^ E/100 g DM. For the DPPH analysis, the results were expressed in scavenging capacity in both IC_50_ and mg TE/100 g DM. For both results, the scavenging capacity increased when compared to the control sample, with the 100% SBF showing the highest values [9].

Costantini et al. [23] tested not only buckwheat but also Tartary buckwheat as ingredients of GFB to verify their impacts on TPC and antioxidant capacity. Gallic acid, 4-hydroxybenzoic acid, p-hydroxybenzoic acid, vanillic acid, protocatechuic acid, syringic acid, salicylic acid, p-coumaric acid, o-coumaric acid, and caffeic acid have all been found in Tartary buckwheat, in addition to four times the amount of flavonoids compared to regular buckwheat [17]. Both breads were made with 100% buckwheat or Tartary buckwheat flour and presented higher TPC than the control bread (wheat-based bread). Tartary buckwheat bread presented a higher TPC (53.3 mg GAE/g dry matter (DM)), while buckwheat bread had 16.5 mg GAE/g DM. According to the authors, the higher TPC for Tartary buckwheat bread is a result of its high rutin content. The authors also evaluated the samples regarding the oxygen radical absorbance capacity (ORAC) analysis. Both samples with Tartary buckwheat had a higher ORAC value than the buckwheat bread, but there was no significant difference among them. 

Ziobro et al. [24] used 10% buckwheat flour to substitute the original flour mix. The TPC analysis was done separately for the crumb and crust. The control bread showed no phenolic compounds in the crumb, while the crust was identified as having 25 mg caffeic acid equivalents (CE)/100 g DM. For the buckwheat bread, the crumb had 64 mg CE/ 100 g DM and the crust had 105 mg CE/100 g DM, showing an increase in the TPC of both parts of the bread. 

The authors also performed the ABTS scavenging activity. Both the crust and crumb of the 10% buckwheat sample showed higher scavenging activity than the control bread, with values of 17.46 ± 0.56 mmol Trolox/g DM for the crust and 13.54 ± 0.47 mmol Trolox/g DM for the crumb. 

Wronkowska et al. [25] used dehulled buckwheat grains to produce buckwheat flour milled and added 10%, 20%, 30%, 40%, and 50% of this flour to substitute the gluten-free flour mix to verify its impact on the TPC of the samples. When compared to the control bread, the buckwheat flour samples showed significantly higher TPC, ranging from 0.42 mg ferulic acid equivalents (FAE)/g fresh matter (FM) to 1.22 mg FAE/g DM, and presented a linear relation between the quantity added and the TPC observed. The lack of literature using this raw material in baking products and using ferulic acid to determine the TPC of samples makes these results difficult to compare. However, it is possible to compare them with other works presented in this review, as shown in the previous section, since the addition of buckwheat flour increased the TPC of the bread, similar to this study. This shows a pattern with the use of buckwheat to enhance the TPC of GFB.

For DPPH analysis using Trolox, all samples showed higher scavenging activity than the control bread, with significant differences among them, except for the breads with 30% and 40% buckwheat, which did not differ significantly. This result is similar to the results for the TPC of the samples already explored, where the samples with 30% and 40% buckwheat showed no significant difference for this specific characteristic.

### 3.3. Millet Flour

Millet, an ancient grain, has a high fiber content, as well as micronutrients and bioactive compounds. In a study by Čukelj et al. [26], 2.5% of coarse or fine proso millet flour was used, and the addition of millet flour resulted in a 50% increase to TPC. The two different types of flour did not show any difference in TPC, suggesting that the form of the millet flour does not affect this specific characteristic.

Banu et al. [27] produced GFB using whole millet flour purchased from the market. The 100% millet bread had a higher TPC compared to the control sample of 100% rice bread. The millet bread had a phenolic content of 180.09 mg FAE/100 g DM and a radical scavenging activity of only 19.24%, which is much lower than the values observed in the study by Pessanha et al. [28], who used a millet cultivar from Brazil but milled the grain before the breadmaking process. The 100% millet bread produced by Banu et al. [27] had an antioxidant capacity of 2.06 mmol Fe^2+^/100 g DM. In contrast, Pessanha et al. [28] produced a 100% millet bread with an antioxidant capacity of 17 mmol Fe^2+^/100 g DM, which is higher than the results found by Banu et al. [27]. These results indicate that the processing and storage time of millet flour can decrease the quantity of bioactive compounds in GFB.

Millet cultivar from Brazil (BRS 1502) was used by Pessanha et al. [28] to produce GFB. Two types of flour were produced: one by milling the grains (RMF) and the other by extruding RMF (PCMF). The samples of 100% RMF, 100% PCMF, and 50% RMF/50% PCMF had TPC values of 1.22, 1.31 and 1.08 µmol GAE/g DM, respectively. All samples showed significant differences from each other. The extruded flour resulted in bread with the highest TPC, indicating that the phenolic compounds are resistant to this processing. Additionally, the extrusion process not only preserves these bioactive compounds but also helps release the phenolics from the grains, increasing the availability of free phenolics in the product. The authors also measured the DPPH scavenging activity in the samples and expressed the results as radical scavenging activity (SFR, %). The PCMF (extruded flour bread) had the highest scavenging activity, followed by the 50% RMF/50%PCMF bread, with the RMF bread having the lowest value. The values ranged from 69.1% to 80.8% for radical scavenging activity. Similarly, in terms of antioxidant capacity (FRAP analysis), the extrusion process resulted in a significant increase in antioxidant capacity for the samples, with the 100% PCMF bread having the highest value of 23.37 mmol Fe^2+^/100 g DM.

### 3.4. Other Cereals and Pseudocereals and Their Combination

Quinoa and amaranth are pseudocereals extremely used in GFB despite their high nutritional content. Quinoa had a significantly higher concentration of p-hydroxybenzoic acid, vanillic acid, p-coumaric acid, and cinnamic acid when compared to whole wheat, corn, and rice. Gallic acid is the main phenolic acid found in quinoa. Amaranth has been found to include several bioactive substances in its seeds and sprouts, including rutin and hydroxybenzoic acid [14,15].

Alvarez-Jubete et al. [9] investigated the impact of amaranth and quinoa on the TPC of GFB. The samples were made with 50% amaranth flour and either 50% or 100% quinoa flour. The amaranth bread only used 50% amaranth flour, as the 100% amaranth flour bread did not have satisfactory functional attributes in preliminary tests. All samples showed a significant increase in TPC compared to the control sample, with the highest value observed in the 100% quinoa bread. Among the pseudocereals tested, the buckwheat samples had the highest TPC. The 50% sample of both flours had higher antioxidant capacity compared to the control bread but lower compared to the 50% buckwheat flour bread in the same study. Similarly, the 100% quinoa flour bread had a higher antioxidant capacity compared to the control bread but lower compared to the sprouted buckwheat flour bread.

Ziobro et al. used the combination of amaranth flour and maize flour in breads at a percentage of 10%. The maize flour bread had the lowest TPC values in both the crumb and crust, with only the crumb having a significantly higher TPC compared to the control bread. Although the amaranth flour bread had a higher TPC compared to the maize bread, it was not higher than the buckwheat bread [24]. 

Sorghum, a common crop in the Gramineae family, is rich in phenolic compounds including phenolic acids, 3-deoxyanthocyanidins, and condensed tannins. Compared to rice, oats, rye, corn, wheat, barley, and maize, sorghum has a more diversified phenolic compound profile and higher phenolic compound concentration. Like other cereals, such as maize and wheat, sorghum’s phenols are mainly found in the bran. The majority of the phenolic acids found in sorghum grains are p-coumaric acid, caffeic acid, cinnamic acid, ferulic acid, gallic acid, salicylic acid, and vanillic acid. Additionally, sorghum has luteolin, apigenin, eriodictyol, and naringenin as its main flavonoids [19,27].

Banu et al. [27] tested the impact of four different flours (rice, quinoa, sorghum, and millet) on the TPC of GFB. Each bread was prepared with 100% of the respective flour to allow for comparison. The bread made with quinoa flour had the highest TPC, while the bread made with rice flour had the lowest. The values for quinoa, sorghum, millet, and rice were 3.98, 3.87, 1.80, and 0.7034 mg FAE/g DM, respectively. Unfortunately, no control bread was made in this case, and the lack of studies using ferulic acid made it difficult to compare the results. The DPPH scavenging activity analysis showed that the rice flour bread had the lowest value (10.5%), while sorghum and quinoa bread had significantly higher values of 35.01% and 32.85%, respectively. Regarding the antioxidant capacity, sorghum flour bread showed the highest TEAC value, followed by quinoa flour bread, millet flour bread, and finally rice flour bread. The authors used a method previously used in one of their former studies, and the results were expressed in µmol Trolox/g DM [27]. 

Brown rice has become increasingly popular in the diets of people with celiac disease or gluten sensitivity, and the germination of these grains has been shown to improve their nutritional value and biologically active compounds. Cornejo et al. investigated the effects of germination of brown rice on the TPC of breads made with this flour. Five different bread recipes were made: the control with brown rice flour without any processing; Pre-GBR, with soaked brown rice; 12 h GBR, with brown rice germinated for 12 h; 24 h GBR, with brown rice germinated for 24 h; and 48 h GBR, with brown rice germinated for 48 h. All breads, including Pre-GBR, had higher TPC, with the 48 h GBR bread reaching up to 1.5 times the TPC of the control bread [29].

Bączek et al. [30] analyzed the impact of combining oat and buckwheat flour on the phenolic content of breads by testing different combinations of both flours. The samples made using buckwheat were OB20% (80% oat flour, 20% buckwheat flour), OB50% (50% oat flour, 50% buckwheat flour), OB80% (20% oat flour, 80% buckwheat flour), and B (100% buckwheat flour). The sample made with 100% buckwheat flour (B) had the highest TPC (1.27 ± 0.02 mg GAE/g DM), followed by OB80% (1.15 ± 0.02 mg GAE/g DM), with the TPC decreasing with the reduction in the percentage of buckwheat flour in the mix (OB50%: 0.82 ± 0.01 mg GAE/g DM; OB20%: 0.38 ± 0.02 mg GAE/g DM). The researchers also measured the phenolic content after in vitro digestion, dividing them into soluble and insoluble fractions. The results showed the same pattern as the breads before digestion, with sample B having the highest phenolic content, followed by OB80%, OB50%, and OB20%. It is important to note that both the soluble and insoluble fractions of the phenolic compounds were higher after digestion. The values ranged from 3.48 ± 0.12 to 5.6 ± 0.01 mg GAE/g DM for the soluble phenolics and from 1.12 ± 0.04 to 1.62 ± 0.02 mg GAE/g DM for the insoluble phenolics.

The results of the FRAP analysis showed that buckwheat flour is a better alternative for increasing the antioxidant capacity in breads compared to oat flour. The sample with 100% buckwheat flour had 5.55 mmol TE/100 g DM, while the sample with 100% oat flour had 2.54 mmol TE/100 g DM. In the breads made with a combination of both flours, the antioxidant capacity increased with the addition of buckwheat flour, but only the OB80% sample showed a significant increase. The authors also performed the analysis after in vitro digestion, which showed that the antioxidant capacity increased in all bread samples, indicating that gastrointestinal conditions may contribute to the release of bioactive compounds and increase the antioxidant capacity of the breads [30].

The inclusion of buckwheat in oat-based breads also generated a significant increase in scavenging activity with the addition of buckwheat. The values increased as the percentage of buckwheat in the GFB increased. These results were observed not only in the raw extract but also in the extracts after digestion, both in the soluble and insoluble fractions [30].

The combination of buckwheat with chia seeds was also tested to improve TPC of breads. Constantini et al. [23] used combinations of either buckwheat and chia seeds or Tartary buckwheat with chia seeds, substituting 10% of the buckwheat flour with chia flour. The sample recipes also included yeast, sourdough starter (type I), and salt. Tartary buckwheat breads (53.3 ± 7.1 mg GAE/g DM) showed significantly higher TPC compared to the breads made with common buckwheat (16.5 ± 1.1 mg GAE/g DM). However, the addition of chia to the bread dough did not affect the TPC of the samples. Even though the researchers used a sourdough starter in this work, they did not analyze its impact on the samples, so this specific characteristic was not explored in relation to the phenolic content of the breads. For Tartary buckwheat bread, chia seeds did not significantly impact the antioxidant capacity, but for common buckwheat, the addition of chia seeds increased this characteristic.

Bel et al. [13] also tested the combination of buckwheat and chia seeds using a mix consisting of 88.2% light buckwheat flour and 9.8% chia flour. The DPPH scavenging activity of this sample was almost double that of the control bread, with 21% scavenging activity while the control had only 12%. The authors also tested the scavenging activity of the premixes, and the baking process had an impact on this parameter, resulting in a 30% loss for the enriched sample.

### 3.5. Algae Powder

Algae have been found to contain a variety of beneficial compounds such as protein, oils, vitamins, minerals, antioxidants, and natural colorants. The antioxidant activity is mainly due to the presence of phlorotannins, a major group of phenolic compounds in brown algae. In a study conducted by Różyło et al. [31], brown alga powder (*Ascophyllum nodosum*) was used as a substitute for a flour mix consisting of white rice flour, corn flour, and millet flour. Different percentages (2%, 4%, 6%, 8%, and 10%) of algae powder were added, with the weight of rice and corn flour decreasing by 1 g for each percentage increase in algae powder. Interestingly, the addition of 2% and 4% algae powder did not significantly affect the TPC of the breads. However, the recipes with 6%, 8%, and 10% algae powder showed an increase in phenolic compounds, suggesting that brown algae can be used as an alternative source for this purpose. The TPC ranged from 3.7 ± 0.24 mg GAE/g DM (lowest percentage) to 4.7 ± 0.35 mg GAE/g DM (highest percentage). The researchers expected the TPC values to be higher in the bread samples due to the high TPC of the alga powder (42.6 mg GAE/g DM). The TPC of the breads after in vitro digestion was determined, and the addition of algae did not significantly alter the TPC. The authors hypothesized that this may be due to the formation of protein–phenolic complexes.

Furthermore, the authors observed a higher chelating activity in samples with algae addition, which was proportional to the amount of additive in the buffer extracts. However, the reduction in EC50 value (half maximal effective concentration) was only observed in samples with 6% or more algae addition. On the other hand, lower levels of algae addition (up to 6%) had a positive impact on the antioxidant bioaccessibility index. Interestingly, the addition of algae did not significantly affect the OH• scavenging activity of the breads [31].

In a study by Nunes et al. [32], *Tetraselmis chuii* algae powder was used as a substitute for a flour mix consisting of buckwheat flour, rice flour, and potato starch. Different percentages (1%, 2%, and 4%) of algae powder were added. The sample with 4% algae showed a significantly higher TPC (0.24 mg GAE/g DM) compared to the control sample (0.11 mg GAE/g DM). However, the increase in TPC was lower compared to the brown algae used in the study by Różyło et al. [31]. The authors suggested that this lower impact may be attributed to the formation of phenolic–protein complexes, indicating that brown algae may be a more effective alternative for this purpose. 

In terms of antioxidant capacity, the addition of 4% *Tetraselmis chuii* algae significantly increased the FRAP (Ferric Reducing Antioxidant Power) analysis, changing from 0.33 mg AAE/g DM in the control bread to 0.47 mg AAE/g DM. This increase in antioxidant capacity was observed even after baking. Similar positive results were also found in the study by Różyło et al., where the addition of *Ascophyllum nodosum* algae powder led to a decrease in EC50 value in the FRAP analysis, indicating an increase in antioxidant capacity. Additionally, the DPPH analysis showed higher scavenging activity in the 4% *Tetraselmis chuii* algae bread (322 mg AAE/100 g DM compared to the control bread (275 mg AAE/100 g DM). These results were consistent with the increase in TPC observed in the enriched samples.

### 3.6. Fruits

The Acerola fruit stands out for its remarkable abundance of beneficial elements, particularly vitamin C and various antioxidants like anthocyanins, flavonoids, carotenoids, and phenolics. These components have been extensively studied and proven to possess strong antioxidant properties. Within the Acerola fruit, notable anthocyanins include cyanidin-3-α-O-rhamnoside and pelargonidin-3-α-O-rhamnoside, as well as quercetin-3-α-O-rhamnoside, kaempferol glycosides, astilbin, and proanthocyanidin. These polyphenols have all undergone extensive testing to determine their valuable functional properties [33].

Bourekoua et al. conducted a study in which they replaced the flour mix in GFB with acerola fruit powder at varying percentages (1%, 2%, 3%, 4%, and 5%). They found that TPC of the acerola bread samples increased with the addition of acerola fruit powder, with the highest value observed in the 5% of flour, measuring 1010 mg GAE/100 g DM [34]. Additionally, the scavenging activity of the bread samples, determined through DPPH analysis, was higher in all samples containing acerola powder compared to the control bread, which had a scavenging activity of 361.4 mg/mL. Even the smallest addition of acerola powder significantly impacted the scavenging activity, increasing it to 231.9 mg/mL, and reaching 67.8 mg/mL in the sample with the highest percentage of powder used [34]. The ABTS radical scavenging activity results also showed a decrease in the EC_50_ value (indicating higher antioxidant activity) with increasing acerola fruit powder addition [34].

Guava (*Psidium guajava*) is a rich source of antioxidants and dietary fiber along with many valuable compounds such as flavonoids, vitamin C, sesquiterpene alcohols, tannins, essential oils, phenolic compounds, and triterpenoid acids. Arslan et al. [35] investigated the use of guava pulp powder in GFB at various percentages (2.5%, 5%, 7.5%, and 10%) to enhance its nutritional value. All bread with guava pulp powder demonstrated higher TPC than the control bread, and the increase was directly proportional to the quantity added. These findings align with those of Nunes-Alves et al. [36], who observed increased TPC in wheat bread with the addition of guava flour, suggesting the potential of this ingredient to boost phenolic compounds in the bread matrix [35,36]. The scavenging activity of the bread samples was also examined, with the results expressed as a percentage. All breads with guava pulp powder exhibited higher scavenging activity than the control bread, and the scavenging activity showed a linear relationship with the amount of guava pulp powder added. Significant differences were observed among all samples, with scavenging activity reaching up to 29.75%, while the control bread displayed 26.47% scavenging activity.

### 3.7. By-Products 

The incorporation of by-products in food products not only reduces food waste but also can increase its polyphenol content, contributing to a more sustainable and healthier food choice. Apple pomace is rich in endogenous polyphenols, such as phenolic acids (especially chlorogenic acid), flavonoids (catechins, epicatechins), and dihydrochalcone (phloridzin) [7]. In a study conducted by Gumul et al., apple pomace was added to GFB samples in three different percentages: 5%, 10%, and 15%. The addition of apple pomace resulted in a significant increase in the TPC of the samples. The TPC of the bread with 15% of apple pomace was found to be the highest at 21.95 mg GAE/100 g (DM), while bread with 5% and 10% of apple pomace had 3.58 and 7.15 mg GAE/100 g (DM), respectively. These results indicate that the TPC of the enriched bread samples ranged from 2.5 to 20 times higher than the control bread [8]. Regarding the antioxidant capacity (mg Trolox/g DM), the apple pomace samples showed a significant increase in the scavenging activity when compared with the control sample, and significant differences among each other, with a linear increase in the scavenging activity with the addition of apple pomace [8].

In another study by Littardi et al. [37], the effects of adding coffee parchment to GFB were tested by TPC. Coffee is known as a stimulant, a property mainly attributed to caffeine; however, the number of chemical compounds identified in this beverage is large, and some of them have many beneficial attributes, such as antioxidant, hepatoprotective, hypoglycemic, and antiviral activities. The production of green tradable coffee beans renders thus several byproducts, depending on the processing method followed. The main byproduct of the dry processing is composed by the skin, pulp, mucilage, and parchment, all together in a single fraction (coffee husks). Wet processing, in contrast, potentially allows for the recovery of the skin and pulp in one fraction (43.2% *w*/*w* from the whole fruit); the mucilage and soluble sugars in a second fraction when fermentation is not used (11.8% *w*/*w*); and finally, the parchment (6.1% *w*/*w*) [38]. When 2% coffee parchment was used in place of the original flour mix, there was no change in the phenolic content, with a value of 1.07 µg GAE/g DM.

Guglielmetti et al. [39] also used coffee by-products, namely silverskin and husk extracts, in approximately 3.4% substitution to the original flour mix. Both materials were effective in increasing the TPC of the GFB compared to the control sample. The GFB with silverskin extract had a higher TPC of 25492 mg CGAE/100 g DM compared to the store-bought flour mix bread (control). The GFB with the husk extract showed a TPC of 12112 mg CGAE/100 g DM. The ABTS scavenging activity was also measured, and the results were expressed in mg chlorogenic acid eq/g freeze-dried sample. Both samples showed a significant increase in scavenging activity when compared to the control bread. The silverskin extract bread showed a scavenging activity of around 280 mg CGA eq/g freeze-dried sample, and the husk extract bread showed around 130 mg CGA eq/g freeze-dried sample.

Krupa-Kozak et al. [40] used broccoli leaf powder to replace 5% of corn starch in the bread, representing approximately 11% of the flour mix. The addition resulted in an increase in the TPC of the bread when compared to the control, with the broccoli leaf powder the bread having 125 mg GAE/100 g DM, almost 50% higher than the control bread (64 mg GAE/100 g DM) [40]. The scavenging activity of the broccoli leaf powder GFB was also significantly higher, with a value of 1.77 ± 0.06 µmol Trolox g/DM compared to the control bread with only 0.13 ± 0.01 µmol Trolox g/DM. When expressed in mg TE/100 g DM, the broccoli leaf powder bread also showed a significant increase (95 µmol TE/100 g DM, a) when compared with the control bread (27 mg TE/100 g DM). 

In another study by Gumul et al., sour cherry pomace extrudates were used to replace 10% of the original flour mix in bread. The extrudates were made with rice flour and 10% or 20% sour cherry pomace at temperatures of 80 °C and 120 °C. The bread with the sour cherry extrudate showed much higher TPC compared to the control bread, with the highest TPC observed in the bread with 20% sour cherry pomace in the extrudate treated at 120 °C. The samples enriched with sour cherry pomace also showed a significant increase in scavenging activity compared to the control samples, although the exact method used and the values expressed were not described [41].

In a study conducted by Bedrníček et al. [42], the effects of onion by-products on the TPC of GFB were investigated. Onion by-products could be used as dietary fiber sources and as antioxidant ingredients due to their phenolic content, reducing at the same time the environmental impact of the tons of onion waste generated [43].

Onion peel and onions that did not meet quality standards were utilized. The onions were either fried (FO), dried (DO), or only the peels were added (OP), with all samples subsequently dried and milled into powder form for use in the bread. A 5% addition of onion by-products was incorporated into all samples, and TPC analysis was conducted before and after baking. The results indicated that all samples with onion by-product addition exhibited higher TPC compared to the control sample. Notably, the baking process did not significantly affect the phenolic content of the bread, suggesting that bread can potentially serve as a vehicle for delivering bioactive compounds to consumers even after heat processing. The study also included FRAP analysis, which revealed a significant increase in antioxidant capacity for the onion by-product breads, ranging from 96 mg TE/100 g DM to 636 mg TE/100 g DM, in comparison to the control sample. The onion peel sample displayed the highest antioxidant capacity, followed by the samples with dry onion and fried onion additions, reflecting a similar trend observed in the TPC analysis.

For the DPPH test, the GFB with the addition of onion by-product showed higher scavenging activity than the control bread, with the onion peel sample showing the highest values for both baked and raw dough. All breads showed a decrease in the scavenging activity after the baking process, with the exception of the onion peel bread, in which the scavenging activity went from 405 mg TE/100 g DM in the raw dough to 470 mg TE/100 g DM in the baked bread. This is explained by the authors as a result of the degradation of quercetin derivatives, allowing it to have a higher antioxidant capacity than when bounded [42].

Seeds are known to be rich in antioxidant compounds, and pomegranate parts are currently being used to enhance this characteristic in food products. Bourekoua et al. [44] utilized a commercially available pomegranate seed powder at various percentages (2.5%, 5%, 7.5%, and 10%) as a substitute for rice flour in GFB production. The breads incorporated with pomegranate seed powder exhibited a significant increase in TPC, with a linear relationship between the amount added and phenolic content. TPC values ranged from 1.29 mg GAE/g DM to 2.47 mg GAE/g DM, and even the lowest addition of pomegranate seed powder resulted in a 46% increase in TPC compared to the control sample. Unfortunately, no other studies were found that employed pomegranate seeds in baked products for comparison.

Bourekoua et al. [44] also evaluated the reducing power of GFB enriched with pomegranate seed powder. Pomegranate (Punica granatum) is native from Iran to northern India and cultivated over the entire Mediterranean region. Pomegranate is a rich source of tannins and other phenolic compounds. By-products of pomegranate can offer a practical and economical source of natural antioxidants that could replace synthetic antioxidants [45]. All samples with the addition of the pomegranate seed powder showed an increase in reducing power compared to the control. The method and results were consistent with those described in the aforementioned section on the study involving moringa. Additionally, pomegranate seed powder was found to enhance the hydroxyl radical (OH•) scavenging activity of GFB. The authors used the method outlined by Su et al., and expressed the results as EC50 value. The formulation with the highest PSP addition demonstrated the greatest impact on scavenging activity.

Lastly, blackcurrant- and strawberry-enriched bread displayed heightened radical scavenging activity relative to the control bread. The sample enriched with 15% strawberry seeds exhibited the highest scavenging activity among all samples. The method employed was the same as described by [46], and the results were expressed in mg Trolox/g DM.

### 3.8. Moringa Leaf

Moringa leaves are packed with various beneficial compounds, including carotenoids, tocopherols, ascorbic acid, phenols, and flavonoids. The Moringa oleifera tree is cultivated for its leaves and seeds, which are used as a fresh vegetable, green manure, plant biostimulant, and biopesticide. Moringa leaf extract contains high amounts of nutrients (N, P, K, Ca, Mg, Fe, Zn, and Cu), antioxidants (ascorbic acid, phenols, carotenoids, proline, enzymes, vitamins A), phytohormones (cytokinins, zeatin, gibberellins, and indole-3-acetic acid), amino acids, vitamins, soluble sugars, and chlorophyll [47]. Bourekoua et al. [48], conducted a study where the leaves were transformed into powder form and incorporated into bread dough at different percentages (2.5%, 5%, 7.5%, and 10%). The strategy significantly enhanced the TPC of the bread, indicating an increase in the presence of these important compounds. 

Agrahar-Murugkar et al. made wheat bread fortified with moringa leaf, and it showed a TPC of 1.5 mg GAE/g DM with the addition of approximately 2.3% of dried moringa leaves replacing the wheat flour. Meanwhile, the GFB produced by Bourekoua et al. showed a TPC ranging from 0.88 to 2.39 mg GAE/g DM. It is possible to notice that the lowest MLP substitution in the GFB showed a lower TPC than the wheat bread, which has a similar MLP added value. This difference may be a result of the type of MLP used, since Bourekoua et al. used a store-bought moringa leaf powder that suffered all the processing and had to endure the storage time, while Agrahar-Murugkar et al. used the fresh moringa leaf and made themselves the MLP, having a fresher material with less loss of its nutritional values. But even with this difference, the values are close, showing that the results of TPC in the GFB are not far from the observed values in other work [48,49].

Bourekoua et al. also observed a noteworthy rise in the ABTS scavenging activity of the MLP gluten-free breads compared to the control bread, with scavenging activity values ranging from 7.51 ± 0.16 to 4.72 ± 0.09 (measured in EC_50_). In contrast, the control sample exhibited a scavenging activity of 9.95 ± 0.21. The results were expressed in EC_50_ and the samples with 2.5% and 5% MLP showed no difference in the scavenging activity when compared to the control bread. The samples 7.5% and 10% MLP had a significant increase in scavenging activity when compared to the other samples and the control bread, but no significant difference among each other [48]. In addition, the MLP samples showed an increase in the reduction in power. The two higher percentages of MLP presented no significant difference among each other. The results were expressed in EC_50_ value [48]. 

### 3.9. Purple and Red Potato

Gumul et al. utilized the pulp (after the starch isolation) from red (Magenta Love) and purple (Violetta) potatoes to replace the flour mix in the GFB recipes at percentages of 5, 7.5, and 10%. The addition of potato pulp resulted in higher TPC in the bread samples compared to the control bread. Among the samples, the bread with 10% purple potato pulp exhibited the highest TPC at 38.71 mg CE/100 g DM. The authors attribute this higher TPC in the purple potato pulp to its elevated content of bioactive compounds [50].

Additionally, Gumul et al. observed an increase in ABTS scavenging activity when adding purple and red potato pulps to the bread. The sweet potato (*Ipomoea potatoes* L.) is considered the fifth most important crop in 40 developing countries, mainly because it is rich in complex carbohydrates, dietary fiber, minerals, and vitamins. Many studies have shown the beneficial health effects provided by sweet potatoes, such as antioxidant, antitumor, hepatoprotective, anti-inflammatory, antidiabetic, antimicrobial, antiobesity, and antiaging activities [51,52]. The scavenging activity values ranged from 3.08 ± 1.22 mg Trolox/g DM in the control bread to as high as 39.4 ± 1.79 mg Trolox/g DM in the bread with 10% red potato pulp. All samples with red potato pulp exhibited higher scavenging activity compared to those with purple potato pulp [50].

Salvador et al. [53] examined two GFB formulations using red potatoes at 37.5% (OP1) and 49% (OP2) percentages. Both formulations displayed a significant increase in TPC in comparison to the control bread, which had a TPC of 0.178 mg GAE/g DM. OP1 exhibited a TPC of 0.790 mg GAE/g DM, while OP2 had a TPC of 0.797 mg GAE/g DM. These values indicate a roughly 300% higher TPC and were attributed by the authors to the presence of phenolic compounds such as anthocyanins and flavonols in red potatoes.

### 3.10. Other Matrices

The study conducted by Sulieman et al. [54] aimed to increase the bioactive compounds in GFB by adding *Agaricus bisporus* powder (ABP) and inulin. Three different percentages of these ingredients (3%, 6%, and 9%) were incorporated into the bread formulation, resulting in six samples. Three samples contained inulin, three contained ABP, and a control bread was included for comparison. The addition of both ingredients significantly increased the TPC of the breads. The ABP bread showed higher quantities of these compounds compared to the inulin bread, which the author attributed to the pigments present in ABP. Moreover, the ABP breads exhibited higher scavenging activity than the control bread. Among the samples, ABPF3 had the highest scavenging activity. The inulin samples exhibited lower EC_50_ values than the control bread, and although they were higher compared to the ABP samples, there was a linear decrease in scavenging activity with increasing inulin content.

Sulieman et al. also analyzed the reducing power of the samples, and the results were expressed as EC_50_ value. All samples, enriched with ABP flour and inulin showed an increase in the reducing power, with the sample with 9% ABP flour presenting the highest value observed [54].

In another study by Conte et al. [55], GFB was supplemented with bee pollen due to its high content of proteins, essential amino acids, vitamins, minerals, and phytochemicals. Bee pollen is a beekeeping product composed of flower pollen, nectar, and bees’ salivary secretions, which is consumed by bees in its fermented form (bee bread) as a source of protein and fat [56]. Different percentages of bee pollen (ranging from 1% to 5%) were substituted for the flour/starch mix in the bread formulation. The addition of bee pollen increased the TPC, soluble phenolic content, and insoluble phenolic content of the bread samples compared to the control bread. The researchers also assessed the bioaccessibility of these compounds, which exhibited an increase ranging from zero in the control bread to 36% in the bread with the highest addition. Notably, as the addition of bee pollen increased, the insoluble/soluble polyphenol ratio decreased, indicating a greater fortification of the bread. This study was pioneering in the use of bee pollen in breads, thus preventing a direct comparison of these results with other works. The antioxidant and scavenging activities of the bread samples also increased with the addition of bee pollen, except for the bread with 1% bee pollen.

The authors also tested the DDPH scavenging activity by measuring the absorbance every 5–10 min until reaching the plateau, in 60 min. For the antioxidant activity, the results were expressed in percentage. The antioxidant activity increased with the addition of the bee pollen, except for the bread with 1% bee pollen. The scavenging activity also increased with the addition of bee pollen [55].

Graça et al. [57] explored the use of yogurt and cheese curd powder as substitutes for gluten-free flour in bread, at percentages of 10% and 20%. Both ingredients significantly increased the TPC of the bread, with higher percentages resulting in greater quantities of phenolic compounds. The TPC of the control bread was 8.5 mg GAE/mg DM, whereas the bread with yogurt and cheese curd ranged from 13.2 mg GAE/mg DM to 15.0 mg GAE/mg DM, representing a 55.3% and 73.0% increase, respectively. The DDPH scavenging activity of the bread enriched with yogurt and cheese curd was also assessed, with the bread containing 20% yogurt demonstrating the highest scavenging activity. All enriched samples exhibited higher scavenging activity than the control bread, which had approximately 2500 mg AAE/100 g DM, while the enriched samples varied from 3500 mg AAE/100 g DM to 5800 mg AAE/100 g DM. The FRAP analysis revealed an increase in the antioxidant capacity of the bread with the addition of yogurt and cheese curd, with no difference observed between the samples with 10% and 20% of each ingredient. Only the bread with 20% cheese curd displayed a significant difference compared to both yogurt breads.

Rózyło et al. [58] investigated the impact of carob fiber on the antioxidant capacity of GFB. Carob fiber contains not only dietary fiber but also polyphenols and tannins. Five different percentages (1% to 5%) of carob fiber were added to the bread formulation. The TPC of the samples was tested after chemical extraction, buffer extraction, and in vitro digestion. For both chemical and buffer-extracted phenolics, the TPC increased with the addition of carob fiber, with a higher content observed for the latter, but there was no correlation between this parameter and the quantity added. For the extracts after the in vitro digestion, the TPC showed much higher levels, which indicates the release of bioactive compounds in the samples. On the other hand, only the control bread and the sample with 1% fiber added showed a higher TPC, while the others had no increase in this characteristic. These results indicate that carob fiber is a raw material with a modest impact on the TPC of breads. 

The authors also used the FRAP analysis to measure the antioxidant capacity of the samples. All samples with the carob fiber enrichment showed a higher antioxidant activity than the control bread, with values ranging from 13.8 EC_50_ mg/mL DM to 17.5 ± 0.61 EC50 mg/mL DM. No correlation between the carob fiber addition and the antioxidant activity was observed. For the scavenging activity, the authors analyzed chemical extracts, buffer extracts, and extracts after an in vitro digestion. Carob fiber is also a material that showed an impact on the chelating activity of Fe^2+^ in GFB. Rózyło et al. used a method described by Guo et al. and the results were expressed in EC_50_ value. For the chemical and digested extracts, all samples showed an increase in chelating activity of Fe^2+^ with the addition of carob fiber. For the buffer extracts, only samples with 1% and 2% fiber addition had a positive impact on this characteristic [58]. 

Paciulli et al. decided to study the impact of the addition of chestnut flour in the antioxidant capacity of GFB. Four different recipes were made: M1 and M2 with two different commercial bread mixes, M1C with 800 g of commercial bread mix 1 and 200 g of chestnut flour (20% CF), and M2C with 900 g of commercial bread mix 2 and 100 g of chestnut flour (10% CF). For both recipes enriched with the chestnut flour, the scavenging capacity has increased when compared with the control breads. The evaluation also was made after 1 and 3 days of storage. For MC2, there was a linear decrease in the scavenging capacity with the increase in storage time. For MC1, on the other hand, there was only a decrease from day 0 to day 1, but there was no significant difference between days 1 and 3, indicating that the highest percentage of chestnut flour addition improves antioxidant capacity and its stability [12]. 

## 4. Conclusions

Efforts to address the deficiencies in gluten-free breads are being made by incorporating various food matrices that are rich in antioxidant compounds. Among these matrices, buckwheat has been extensively studied and has shown promising results in terms of bioactive compounds. The use of Tartary-variety or wholegrain buckwheat further enhances these compounds. Buckwheat has been combined with various cereals and pseudocereals in numerous studies, and in most cases, the positive outcomes in terms of enhancing polyphenolic content are attributed to the presence of buckwheat grains. Amaranth, sorghum, and acorn are other examples of good matrices to enhance the TPC of GFB.

The addition of different by-products can effectively increase the bioactive compounds and antioxidant activity in bread products, making it a viable strategy to enhance nutritional value while reducing costs. However, it is important to consider the sensory response of consumers when enriching these products, as matrices with high phenolic content can affect the taste, color, and aroma of breads. Surprisingly, only 17 out of the 36 papers reviewed conducted sensory analysis on their samples.

Although it is known that heat processing releases phenolic compounds from the cell wall of buckwheat grains, there is limited information on the behavior of bioactive compounds and antioxidant activity during the breadmaking process. Furthermore, there is a lack of studies evaluating the bioaccessibility of these compounds and their impact on human health. In vivo and clinical studies are needed to provide a better understanding of how bioactive compounds function after consumption.

## Data Availability

Data are contained within the article.

## Appendix A

**Table A1:** Summarizing the effects of different matrices on the overall quality of gluten-free bread.

Authors	Food Matrix and Percentage Added	Antioxidant Analyses	Reagents	Results Expressed	Sensory Analysis	Effects
BOUREKOUA et al., 2021 [34].	Acerola: 1, 2, 3, 4 and 5%	TPC	Gallic Acid	mg GAE/g DM	Yes	Increase in volume with increase in acerola level; increased size and area fraction of cells; decreased firmness and chewiness by increased springiness; increased antioxidant activity; good acceptance up to 3% acerola addition.
		DPPH	Gallic Acid	EC50 value		
		ABTS	Gallic Acid	EC50 value		
		RED	Gallic Acid	EC50 value		
SKENDI et al., 2018 [20].	Acorn 5, 15 and 25%	TPC	Gallic Acid	mg GAE/g DM	No	Color improvement; increase in TPC; decrease in volume and increase in hardness with increase in acorn levels.
MARTINS et al., 2020 [11].	Acorn 25 and 35%	TPC	Gallic Acid	mg GAE/g DM	Yes	23% of addition increased bread volume; darker color of crust/crumb; increase in antioxidant capacity; improvement in sensory characteristics.
		ABTS	Trolox	mmol TE/g DM		
		DPPH	Trolox	mmol TE/g DM		
		FRAP	Trolox	mmol TE/g DM		
SULIEMAN et al., 2019 [54].	*Agaricus bisporus* polysaccharide flour: 3, 6 and 9%	TPC	Gallic Acid	mg GAE/g DM	No	Higher levels of volatiles; increase in antioxidant capacity.
		ABTS	ABTS	EC50		
		DPPH	DPPH	EC50		
		Reducing power	-	EC50		
RÓŻYŁO et al., 2017 [31].	Brown algae: 2, 4, 6, 8 and 10%	TPC	Gallic Acid	mg GAE/g DM	Yes	Larger volume with 4% algae; increased antioxidant capacity; high bioaccessibility of antiradical compounds (in vitro); unpleasant taste over 4% addition; firmness and staling decreased, elasticity incresed with algae increase.
		ABTS	Trolox	EC_50_ mg DM/mL		
		CHEL	Unclear	EC_50_ mg DM/mL		
		FRAP	Unclear	EC_50_ mg DM/mL		
NUNES et al., 2020 [32].	Algae 1, 2 and 4%	TPC	Gallic Acid	mg GAE/g DM	Yes	4% algae bread with increase in volume, decrease in firmness, increase in TPC and antioxidant capacity, but with lowest scores in sensory analysis.
		DPPH	Ascorbic Acid	mg AAE/g DM		
		FRAP	Ascorbic Acid	mg AAE/g DM		
ALVAREZ-JUBETE et al., 2010 [9].	Amaranth, quinoa, buckwheat	TPC	Gallic Acid	mg GA/g	No	Highest TPC and antioxidant capacity for buckwheat bread; sprouting increased antioxidant capacity.
		FRAP	Trolox	mg T/g DM		
		DPPH	Trolox	mg T/g DM		
GUMUL; ZIOBRO, 2021 [8].	Apple pomace: 5, 10 and 15%	TPC	Rutin	mg rutin/100 g DM	Yes	5% enriched bread with highest scores in sensory analysis and TPC; increase in TPC and antioxidant capacity with increased apple pomace added.
		ABTS	Trolox	mg Trolox/g DM		
CONTE et al., 2020 [55].	Bee pollen: 1, 2, 3, 4 and 5%	TPC	Gallic Acid	mg GA/g DM	No	Breads with >2% pollen presented: Increase in proteins, minerals, soluble and bioaccessable polyphenols, total carotenoids, and antiradical activities
		DPPH	DPPH	%		
KRUPA-KOZAK et al., 2021 [40].	Broccoli leaf	TPC	Gallic Acid	mg GA/g DM	No	Increase in proteins and minerals; improved specific volume and baking loss; increase in antioxidant potential; inhibitory activity against advanced glycation end-products.
		ABTS	Trolox	µmol T/g DM		
		DPPH	Trolox	µmol T/g DM		
		PCL	Trolox	µmol T/g DM		
SAKAC et al., 2011 [22].	Buckwheat: 10, 20, and 30%	TPC	Gallic Acid	mg GA/g DM	No	Increase in antioxidant properties with increase in buckwheat addition; whole buckwheat flour breads presented higher antioxidant properties than light buckwheat flour breads.
		Reducing power	-	IC_50_ mg/mL		
		DPPH	DPPH	IC_50_ mg/mL		
		Chelating activity Fe^2+^	Fe^2+^	IC_50_ mg/mL		
ZIOBRO et al., 2016 [24].	Buckwheat, amaranth, maize: 10%	ABTS	Trolox	mmol T/kg DM	No	Buckwheat decreased hardness and increased cohesiveness during storage, increased TPC and antioxidant capacity; amaranth improved nutriotional characteristics.
WRONKOWSKA et al., 2010 [25].	Buckwheat: 10, 20, 30 and 40%	TPC	Ferulic Acid	µmol FA/g DM	Yes	Highest percentage showed highest antioxidant capacity and sensory quality.
		ABTS	Trolox	µmol T/g DM		
		DPPH	Trolox	µmol T/g DM		
		Reducing capacity	Trolox	µmol T/g DM		
BEL et al., 2021 [13].	Buckwheat + chia: 90:10	DPPH	DPPH	%	No	Light buckwheat flour breads with chia seeds and xanthan gum had higher air fraction, alveolar area and lightness; breads presented higher protein, crude fiber, ash, polyunsaturated fatty acid content and antioxidant activity than the commercial breads.
RÓZYŁO et al., 2017 [58].	Carob fiber: 1, 2, 3, 4 and 5%	TPC	Gallic Acid	mg GA/g DM	Yes	Improvement in color, volume, springiness and decrease in hardness with carob fiber addition; increase in antioxidant capacity with 1% and 2% carob added breads; 2% bread with better acceptance in sensory analysis.
		ABTS	ABTS	EC_50_ mg mL DM		
		Chelating activity Fe^2+^	Unclear	EC_50_ mg mL DM		
		FRAP	Unclear	EC_50_ mg mL DM		
PACIULLI et al., 2016 [12].	Chestnut: 10 and 20%	DPPH	Trolox	mmol/L Trolox/g	No	Browning in color; lower bulk volume and larger crumb holes; decrease in crumb cohesiveness and resilience; faster staling; higher antioxidant capacity; 20% enriched bread with an increase in fiber content.
LITTARDI et al., 2020 [37].	Coffee: 2 and 4%	TPC	Gallic Acid	mg GA/g DM	Yes	Positive sensory evaluation with 2% enrichment; 4% enriched bread with bitter taste; improved color; increased antioxidant capacity; reduced HMF content.
		DPPH	DPPH	%		
GUGLIELMETTI et al., 2019 [39].	Coffee: 2.5%	TPC	Chlorogenic Acid	mg CGA/g DM	Yes	Decrease in bioaccessibility of sugars; increase in antioxidants; good consumer acceptance.
		ABTS	Chlorogenic Acid	mg CGA/g DM		
		ABTS	Trolox	mg Trolox/g DM		
GRAÇA et al., 2020 [41].	Curd and yogurt: 10 and 20%	ABTS	Trolox	mg Trolox/g DM	No	20% curd bread with 35% reduction in glycaemic response; curd bread with better reducing power; yogurt bread with better radical scavenging capacity; increased antioxidant activity with both enrichments; higher MMP-9 inhibition activity.
		TPC	Gallic Acid	mg GA/g DM		
		DPPH	Ascorbic Acid	mg AA/mg DM		
		FRAP	Ascorbic Acid	mg AA/mg DM		
ARSLAN et al., 2017 [35].	Guava: 2.5, 5, 7.5 and 10%	TPC	Gallic Acid	mg GA/g DM	Yes	Increased crude fiber; increased TPC; volume increased, and hardness decreased; 5% enriched bread with the best sensory results.
		DPPH	DPPH	%		
PESSANHA et al., 2021 [28].	Millet, precooked flour (PCMF) and wholegrain flour (RMF): 100% PCMF, 100% RMF and 50:50 PCMF and RCMF	TPC	Gallic Acid	µmol GA/g DM	No	Extrusion increased antioxidant capacity and enzymatic inhibition.
		DPPH	DPPH	% SFR		
		FRAP	Trolox	µmol T/g DM		
BANU; APRODU, 2020 [27].	Millet, quinoa, sorghum, rice: 100% of the whole grain flours	TPC	Ferulic acid	mg FA/g DM	No	Quinoa bread with highest content of protein, ash, fat, total dietary fiber, resistant starch content and TPC; quinoa and rice breads with higher specific volume and lower crumb firmness; sorghum bread with highest antioxidant capacity.
		DPPH	DPPH	%		
		FRAP	Trolox	µmol Fe^2+^/g DM		
		TEAC	Trolox	µmol T/g DM		
BOUREKOUA et al., 2018 [22].	Moringa leaf: 2.5, 5, 7.5 and 10%	TPC	Gallic Acid	mg GA/g DM	Yes	More than 2.5 % addition resulted in reduced specific volume; hardness and chewiness decrease in 2.5 and 10% addition; 2.5% bread with best sensory acceptance; lightness of crust and crumb decreased; TPC and antioxidant capacity increased; 5% bread with optimal conditions.
		DPPH	DPPH	EC_50_ mg DM/mL		
		ABTS	ABTS	EC_50_ mg DM/mL		
		Reducing capacity	Unclear	EC_50_ mg DM/mL		
BĄCZEK et al., 2020 [30].	Oat and buckwheat: 100% each, OB20% (20% buckwheat), OB50% (50% buckwheat), OB80% (80% buckwheat)	TPC	Gallic Acid	mg T/g DM	No	Oat bread with lowest glycemic index; phenolic compounds present even after digestion; oat-buckwheat breads are good source of TPC and high antioxidant capacity.
		ABTS	Trolox	µmol T/g DM		
		FRAP	Trolox	µmol T/g DM		
BEDRNÍČEK et al., 2020 [42].	Fried onion (FO), dried onion (DO), and onion peel (OP) in 5%	TPC	Gallic Acid	mg GA/g DM	Yes	Increase in antioxidant activity, TPC and flavonol content; quercetin precursors released quercetin during baking; consumption of OP bread increase antioxidant activity of consumers’ blood; no differences in sensory analysis from control bread.
		DPPH	Trolox	mg T/g DM		
		FRAP	Trolox	mg T/g DM		
BOUREKOUA et al., 2018 [44].	Pomegranate: 2.5, 5, 7.5, and 10%	TPC	Gallic Acid	mg GA/g DM	Yes	Increase in specific volume and springiness; decrease in hardness and chewiness; decrease in lightness and yellowness, increase in redness of crust and crumb; increase in TPC and antioxidant capacity; optimum level of 7.5% according to sensory evaluation.
		DPPH	DPPH	EC_50_ mg DM/mL		
		ABTS	Unclear	EC_50_ mg DM/mL		
		Reducing capacity	Unclear	EC_50_ mg DM/mL		
GUMUL et al., 2020 [50].	Red and purple potatoes	TPC	Catechin	mg catechin/100 g DM	Yes	7.5% red potato bread with higher TPC, fiber content and antioxidant activity, low levels of acrylamide and good physical and sensory characteristics.
		ABTS	Trolox	mg TE/g DM		
CORNEJO et al., 2015 [29].	Rice germinated for 0, 12, 24, and 48 h, 100% rice flour	TPC	Gallic Acid	mg GA/g DM	No	48 h bread with higher contents of protein, lipids, bioactive compounds, and antioxidant activity, reduced phytic acid content and glycaemic index, slight decrease in in vitro protein digestibility.
		ORAC	Trolox	mg T/g DM		
GUMUL; KORUS; ZIOBRO, 2020 [41].	Sour cherry pomace: 10 and 20%	TPC	Catechin	mg catechin/kg	No	Increase in bioactive compounds and antioxidant activity.
		ABTS	Trolox	μM T/kg DM		
		TPC	Gallic Acid	mg GA/g DM		
		DPPH	Butylated hydroxyanisole	μmol/g dwt or %		
KORUS et al., 2012 [46].	Strawberry and blackcurrant seeds: 5, 10, and 15%	ABTS	Trolox	mg T/g DM	Yes	Modification of viscoelastic properties, reduced values of consistency coefficients and flow indices; decreased hardness; decrease in L * and B * and increase in a * color parameters; no negative impacts in sensory analysis; increase in dietary fiber, protein, and polyphenols.
COSTANTINI et al., 2014 [23].	Tartary buckwheat/buckwheat and chia: 100% buckwheat flour and 10% addition of chia in buckwheat chia breads	TPC	Gallic Acid	mg GA/g DM	No	Chia and Tartary buckwheat bread presented higher protein content, insoluble dietary fiber, ash, and alpha-linolenic acid, and lower carbohydrate content; use of tartary buckwheat improved antioxidant capacity and flavonoids content.
		TFC	Rutin	mg R/g DM		
		FRAP	Fe^2+^	mmol Fe^2+^/g DM		
		ORAC	Gallic Acid	mmol GA/g DM		

* Studies used, analyses made and results found.
